# Multiple Cephalic Malformations in a Calf

**DOI:** 10.3390/ani10091532

**Published:** 2020-08-30

**Authors:** Di Muro G., Cagnotti G., Bellino C., Capucchio M.T., Colombino E., D’Angelo A.

**Affiliations:** Department of Veterinary Science, University of Turin, Largo Paolo Braccini 2-5, 10095 Grugliasco (TO), Italy; giorgia.dimuro@unito.it (D.M.G.); claudio.bellino@unito.it (B.C.); mariateresa.capucchio@unito.it (C.M.T.); elena.colombino@unito.it (C.E.); antonio.dangelo@unito.it (D.A.)

**Keywords:** malformation, holoprosencephaly, hydranencephaly, median cleft face syndrome, bovine

## Abstract

**Simple Summary:**

This case report describes an unusual congenital cephalic malformation involving both the craniofacial structures and the central nervous system in a calf referred to the Veterinary Teaching Hospital, Department of Veterinary Science of Turin (Italy). In the study, we describe the clinical and neurological examination, the analysis conducted, and the evidences at post-mortem evaluation, in order to illustrate a severe congenital abnormality that has never been reported in calves before.

**Abstract:**

Congenital malformations of the central nervous system (CNS) can affect the CNS alone or the CNS and craniofacial structures. Here, we report an unusual and complex congenital cephalic malformation observed in a 3-day-old male crossbreed calf. Clinical examination disclosed a dome-shaped cranial vault, a flat face with a short snout, a median cleft lip, and increased intraorbital distance. The frontal region of the head was remarkable for a fluctuant, sac-like protrusion covered with haired skin. Neurologic findings suggested a multifocal intracranial lesion affecting the prosencephalon and the central vestibular system. While pathological and histopathological findings posited for a presumptive diagnosis of either hydranencephaly or holoprosencephaly associated with multiple congenital facial abnormalities, not all the findings could be definitely attributed to either of the two encephalic malformations alone. To our knowledge, a similar combination of severe congenital abnormalities affecting both the CNS and the craniofacial structures has not been reported in calves to date.

## 1. Introduction

Congenital defects are structural or functional abnormalities that result from errors arising during embryonic development and are present at birth [1]. While reliable data on the incidence of congenital anomalies in domestic animals are not available, a frequency of about 3–4% is estimated in cattle [2]. Such anomalies can range from purely biochemical abnormalities to gross anatomical defects and can include disorders of function, metabolism, and behavior [2]. Congenital malformations of the central nervous system (CNS) can affect the CNS alone or the CNS, the craniofacial structures, and the vertebral column; they are attributable to genetic or environmental causes or to environmental–genetic interactions [3]. This case report describes an unusual, complex congenital cephalic malformation observed in a male crossbreed calf.

## 2. Materials, Methods, and Results

### 2.1. Case Description and Clinical Investigations

**Figure 1:**
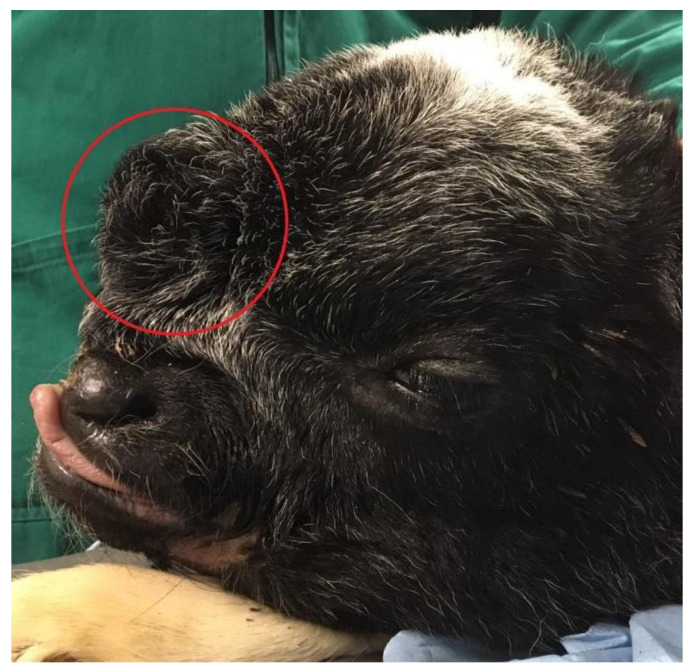
Profile view of calf’s head. The red circle indicates the fluctuant, sac-like protrusion covered with haired skin (suspected meningocele).

A 3-day-old male crossbreed calf was referred to the Mobile Clinic Service, Veterinary Teaching Hospital (VTH), Department of Veterinary Science of Turin (Italy) because of an abnormal facial conformation and inability to stand on forelimbs and hindlimbs since birth. No complications were reported during gestation or birth, and the dam had given birth to other normal calves previously. Clinical examination disclosed a dome-shaped cranial vault. The face was flat, with a short snout, a median cleft lip, and an increased intraorbital distance clearly suggesting hypertelorism. The frontal region of the head was remarkable for a fluctuant, sac-like protrusion (5 × 7 cm) covered with haired skin (Figure 1). The rim of the underlying cranial bone defect could be felt around the base of the sac-like protrusion on palpation.

Neurologic examination performed by a board-certified neurologist (ADA) revealed a mildly obtunded mental status. The calf demonstrated intermittent left head tilt, inability to stand unassisted, and non-ambulatory tetraparesis, when supported, with only minimal voluntary movements. Proprioception and postural reactions were absent on all four limbs. Cranial nerve examination revealed a bilaterally absent menace reaction. Nostril sensation was diminished on stimulation of the right nostril. Physiological nystagmus was absent/severely reduced bilaterally, whereas abnormal positional jerk nystagmus changing in the direction was present bilaterally. Positional ventro-lateral strabismus of the left eye was noted. Spinal reflexes were normal on all four limbs, and palpation of the vertebral column elicited no pain. Based on the findings of the neurologic examination, a multifocal intracranial lesion affecting the prosencephalon and the central vestibular system was suspected. The severity of the gait deficits could be related to a brainstem localization without involvement of the ascending reticular activating system or to an additional cervical spinal cord localization. A congenital malformation was suspected based on the animal’s age, history, and clinical presentation.

Complete blood count (CBC) and biochemistry profile were unremarkable. A cerebrospinal fluid (CSF) sample collected by lumbosacral puncture under general anesthesia was colorless and clear at macroscopic evaluation; the CSF analysis was normal. Soon after CSF taping, the calf was euthanized because of poor prognosis and a post-mortem examination was carried out.

### 2.2. Gross Examination and Histopathology

At necropsy, the calf showed a dome-shaped cranial vault with a 4-cm long cranioschisis along the midline at the level of the frontal bones. Upon dissection, the frontal sac-like protrusion was filled with CSF and internally lined with meningeal tissue, indicating a diagnosis of meningocele. The intraorbital distance was increased (hypertelorism), although both eyes were normally developed. Moreover, the rostral maxilla was shorter than the mandibula (prognathism); a complete median cleft lip and maxilla without cleft palate was present. The nose appeared abnormal, with a broad base and a bifid nasal tip. The single, rudimental nasal cavity identified after head sectioning opened rostrally into the oral cavity through the upper jaw defect. The abdominal and thoracic organs were macroscopically normal. The vertebral column was normal. On gross examination of the CNS, both fresh and after formalin fixation, the cerebral hemispheres, actually consisting in a single cerebral sphere, appeared as a thin, irregular layer of nervous tissue adherent to the skull bones. The convolutional pattern of the gyri was abnormal and slightly smooth. There was massive bilateral symmetrical loss of brain parenchyma; the frontal and parietal cortex and the olfactory bulbs and tracts were absent (olfactory aplasia). The cribriform plate of the ethmoid bone was also absent with a direct communication between the calvaria and the single nasal cavity. A single, dilated ventricle, presumably derived from the confluence of the two lateral ventricles, occupied nearly the entire intracranial space and was filled with abundant CSF. A rudimental dural septum (falx cerebri) was attached to the ridges of the occipital bone caudally. The corpus callosum, fornix, rostral commissure, and septum pellucidum were absent. Multifocal vestiges of nervous tissue were irregularly distributed, particularly on the floor of the cerebral hemispheres on both sides of the diencephalon. The rostral diencephalon was undersized and incompletely organized, whereas the organization of the caudal fossa was preserved: no gross abnormalities were observed in the pons, cerebellum, and cervical spinal cord.

**Figure 2:**
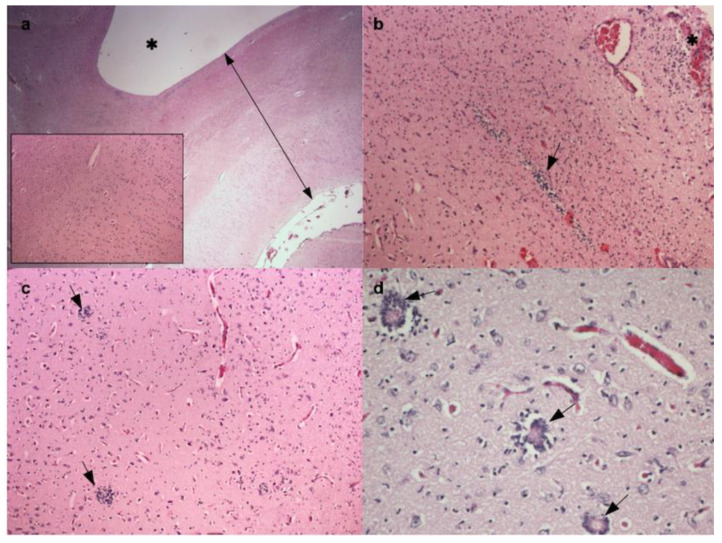
**Histopathology.** (**a**) Brain. Cerebral parenchyma reduced to a thin layer of white and grey matter covered by meninges (black line) and surrounding a voluminous dilated ventricle (*). The inset shows the maintained histological organization. Hematoxylin and eosin (H&E), 20× (**b**) Brain. Cerebral parenchyma with multifocal hemorrhages, especially in the periventricular area (*), and multifocal small clusters of stem cells (black arrow) in subventricular zone. H&E, 100× (**c**) Brain. Cerebral parenchyma with multifocal clusters of undifferentiated cells into the gray matter (black arrows). H&E, 100× (**d**) Brain. Diencephalon with multifocal small rudimental ventricles (black arrows). H&E, 200×.

Brain and cervical spinal cord were routinely processed for histology. The tissues were fixed in 10% formalin phosphate-buffered solution and stained with hematoxylin and eosin (H&E). Selected sections of telencephalic and diencephalic parenchyma were also immunohistochemically stained with antibodies against glial fibrillary acidic protein (GFAP; rabbit anti-cow GFAP; 1:2000 dilution; code number Z0334; Dako, Agilent Technologies Italia S.p.A. Bioscienze ed Analisi Chimica, Cernusco sul Naviglio, Milano, Italy), neuron-specific enolase (NSE; Mab antihuman; 1: 200 dilution; clone BBS/NC/VI-H14; Dako, Agilent Technologies Italia S.p.A. Bioscienze ed Analisi Chimica, Cernusco sul Naviglio, Milano, Italy), and vimentin (Mab antihuman; 1:50 dilution; clone V9; code number MO725; Dako, Agilent Technologies Italia S.p.A. Bioscienze ed Analisi Chimica, Cernusco sul Naviglio, Milano, Italy) followed by the Vectastain ABC-AP Kit Universal (Vector Laboratories, Burlingame, CA, USA). After heating sections at 98 °C for 25 min in citrate buffer for antigen retrieval, 3% *v/v* H_2_O_2_ was added for 5 min to quench endogenous peroxidase activity. Primary antibody was added for 2 h in a humidified chamber at 4 °C, and biotinylated link antibody and peroxidase-labeled streptavidin were added for 10 min followed by 3,3′-diaminobenzidine tetrahydrochloride (DAB). Nuclei were counterstained with hematoxylin. Positive controls were represented by bovine brain sections, and negative controls were carried out by omitting the primary antibodies. Microscopically, the cerebral parenchyma was reduced to a thin layer of white and gray matter covered by the meninges. The histological organization of the cortex was generally maintained (Figure 2a), but newly formed hyperemic blood vessels were disseminated throughout. Multifocal hemorrhages, particularly in the periventricular area, and small multifocal clusters of stem cells in the subventricular zone (Figure 2b) and in the cerebral gray matter (Figure 2c) were present. Scattered phenomena of white blood cells vessels margination in the gray matter and multifocal slight mononuclear cells infiltrates in the overlying meninges were also detected. Histological sections of the diencephalon revealed poor differentiation between the gray and the white matter, which was sometimes mixed with small, rudimental multifocal neuronal nuclei of different size, consistent with the absence of thalamic cleavage. Small rudimental ventricles covered by ependyma (Figure 2d) and disseminated small blood vessels were also observed at this level.

The cerebellum and caudal brainstem had a normal histological structure, whereas the cervical spinal cord had no clear separation between the white and the gray matter. The borders of the dorsal and the ventral horns were ill-defined, scattered neurons were present in the white matter, and the nervous tracts were irregular in shape and spatial orientation.

Immunohistochemistry revealed disseminated NSE positivity of neuronal cells (dispersed or organized in nuclei), indicating cell maturity. Glial cells (astrocytes) had diffuse positivity for GFAP. Multifocal scattered positivity for vimentin was detected in the subependymal area in particular.

A spleen sample taken during necropsy and submitted for the detection of Schmallenberg virus, Bluetongue virus, and bovine viral diarrhea virus (BVDV) by reverse-transcription rtPCR assay tested negative for all three.

Based on the pathological and histopathological findings, a presumptive diagnosis of either hydranencephaly or alobar holoprosencephaly associated with multiple, congenital facial abnormalities was established, though not all the findings could be attributed to either of these two encephalic malformations alone (Table 1).

## 3. Discussion

The neuroanatomic findings and distribution of lesions attributable to malformations of both the face and the CNS in this calf are not easy to classify and trace to a specific condition. Computed tomography/magnetic resonance, which would have helped in defining the morphology of these malformations that notoriously change post-mortem [6], were not performed for financial reasons. The craniofacial abnormalities identified at clinical evaluation and necropsy can suggest a median cleft face syndrome. This syndrome describes a congenital failure of fusion of the facial process during fetal development; it may involve both soft and hard tissue of the face and can have diverse phenotypic manifestations [1,2]. In human medicine, median cleft face syndrome was first described by DeMyer and colleagues in 1967. They identified as phenotypic features the presence of ocular hypertelorism associated with six other median facial defects: a low V-shaped frontal hairline, cranium bifidum occultum, median cleft nose, median cleft upper lip and premaxilla, median cleft palate, and primary telecanthus. Moreover, four facial types are distinguished according to the defects and their degree of severity [7]. The syndrome has also been reported in calves. Moritomo et al. morphologically described a series of 13 calves with cleft face. According to the cleft type and site, four groups of malformation were distinguished: median cleft lip and jaw (CLJ); median cleft lip, jaw, and palate (CLJP); lateral CLJ; and cleft palate (CP), including unilateral and bilateral type. Median CLJ was characterized by clefts extending to the dorsal portion of the nose until the subcutaneous tissues or to the labial portion. When the cleft was longer and extended also vertically (creating a bifid nose), as well as to the median lip and the rostral part of the palate, a condition of CLJP was defined. Lateral CLJ referred to the presence of bilaterally symmetrical lip clefts between the nostrils and the mouth. Finally, CP included clefts of the hard and soft palate, which were either unilateral or bilateral. A number of systemic malformations and clinical signs were reported to be associated with these syndromes [8]. While the necropsy findings of this calf fit within the cleft lip, jaw, and palate (CLJP) group, the meningocele and the severe brain malformations detected in this calf were neither reported nor described in Moritomo’s paper [8].

A more recent classification for median cleft face syndrome in veterinary medicine was devised by Reinartz and colleagues in 2015. According to this system, defects can involve the upper lip, jaw, palate, and nose alone or combined and can be classified as unilateral on the right or left side, median, or bilateral, based on the morphology and location in the rostral face. In addition, midline facial malformations are further subdivided in syndromic or non-syndromic depending on the presence or not of abnormalities in other organs [9]. Congenital facial malformations are often associated with brain malformations, which is probably due to the close correlation between the embryological development of brain, epidermis, and skull [4,10,11].

In 1975, DeMyer and colleagues investigated whether the observation of the craniofacial syndrome could predict the underlying brain anomalies [10]. Their study was focused primarily on the eye position within the face; they found that ocular hypotelorism combined with other median plane facial anomalies due to defects in the intermaxillary segment is usually correlated with very severe brain malformations, such as holoprosencephaly [10]. Differently, ocular hypertelorism associated with median cleft due to failure in the apposition of the frontonasal prominence along the median facial plane is typical of so-called median cleft face syndrome or frontonasal dysplasia (FND), and it is usually associated with normal or mild brain malformations [10]. In human medicine, some syndromic forms of FND are associated with brain malformations (e.g., corpus callosum agenesis, periventricular nodular heterotopia, interhemispheric lipoma, hydrocephalus) that usually cause mild or no intellectual disability [12]. In veterinary medicine, some mild brain malformations associated with median cleft face syndrome have been reported in calves, including cerebellar hypoplasia, hypoplasia of corpus callosum, and an unequal development of cerebral hemispheres [8].

Frontal meningoencephalocele has rarely been reported in human patients with median cleft face syndrome because the fusion defect of the two halves of the frontal bone is not sufficient to cause the protrusion of normal meninges and brain. Active pressure needs to be present to cause these organs to bulge out [10]. In human medicine, one characteristic associated with meningoencephalocele is agenesis of the third ventricle resulting from absence of thalamic cleavage, which blocks CSF flow and increases pressure within the abnormal brain [13].

However, the pathological findings of the brain in this calf are not completely consistent with the above definition of FDN. Histopathology disclosed a severe brain malformation characterized by thinning of the cerebral cortex, absence of several lobes of the cerebral hemispheres (i.e., frontal, parietal, and olfactory lobes), absence of the corpus callosum, and confluence of the two lateral ventricles, with a huge compensatory hydrocephalus. The presence/absence of CSF flow obstruction was impossible to confirm. These findings suggest hydranencephaly, a cerebral abnormality in which the cerebral cortex is reduced to a thin, neuroparenchymal, and meningeal membrane, usually resulting from the viral destruction of germinal layer cells and differentiated neopallial neurons. These two processes produce aplasia and atrophy of the cerebral hemispheres, respectively, especially in their dorsal, medial, and caudal aspects, resulting in their replacement by two fluid-filled sacs containing the two dilatated lateral ventricles with compensatory hydrocephalus and absent ependymal layer [2,4]. However, by definition, hydranencephaly involves only the neopallial structures of the cerebral cortex, while paleopallium, archipallium, diencephalon, mesencephalon, pons, and cerebellum are typically spared [4]. Only cerebellar lesions, such as cerebellar hypoplasia and atrophy, are present in some cases [5]. Moreover, the cranial cavity is usually normal in size and shape [4,5]. In cattle, hydranencephaly is often caused by intrauterine viral infection (e.g., Bluetongue virus, BVDV, Shmallenberg virus, Akabane virus, Aino virus), and the degree of lesion severity depends on the viral agent and the point during gestation when the infection occurred [14]. Sometimes, evidence for an inflammatory and/or ischemic peripheral process with lymphocytic perivascular cuffs, gliosis, and the mineralization of necrotic tissues is observed histologically in the surviving brain parenchyma [5,15]. In this calf, signs of inflammation of the cerebral parenchyma were identified together with disseminated newly formed small blood vessels in the cerebral cortex.

More severe abnormalities of the paleopallial structures (e.g., absence of olfactory bulbs, peduncles, and piriform lobe cortex) and of the diencephalon (e.g., incomplete thalamic cleavage) are typical of holoprosencephaly (HPE) [5]. HPE is a sporadic malformation encountered in domestic animals; it has been described in calves only twice to date [16,17], while it is more common in lambs [4], especially in Border Leicester lambs in which an autosomal recessive transmission has been identified as cause [18]. HPE is characterized by an incomplete midline division of the prosencephalon during embryological development, which results in a single telencephalic vesicle accompanied by absence of the rostral commissure and corpus callosum, septum pellucidum, septal nuclei, and olfactory system [4,5]. Different types of HPE have been classified based on the degree of severity that includes lobar, semilobar, and alobar forms: in lobar HPE, the interhemispheric fissure is present along nearly the entire midline, but the most inferior portions of the frontal lobes are fused; in semilobar HPE, there is only a partial separation between the two cerebral hemispheres, with interhemispheric fissure and falx cerebri that may be present posteriorly; finally, the most severe form is alobar HPE, in which there is a single midline forebrain with a primitive monoventricle often associated with a large dorsal cist [19,20,21,22]. There are various analogies between the features of alobar holoprosencephaly and the findings from this calf in which the facial abnormalities seem more consistent with those typical of holoprosencephaly, except for the hypertelorism. Furthermore, the absence of the longitudinal cerebral fissure that separates the two cerebral hemispheres, a hallmark feature of holoprosencephaly, is missing in the present case.

There was a poor distinction between the white and the gray matter of the cervical spinal cord. These spinal cord abnormalities have never been reported before in calves based on Cho and Kisipan papers [14,15]. Indeed, spinal cord abnormalities reported in cattle with intrauterine viral infection (BVDV, Shmallenberg virus, Akabane virus, Aino virus) are different. They are bilateral symmetrical spinal lesions characterized by hypomyelination, axonal dysplasia, Wallerian-type degeneration, and neuron loss in the spinal cord ventral white matter and the ventral horns of the gray matter [14]. Moreover, the calf of the present study tested negative for BVDV, Blue tongue virus, and Shmallenberg virus at rtPCR. Akabane virus and Aino virus were not investigated because they are not currently reported in Italy [22,23,24].

The origin of congenital disorders can be traced in the interaction between the genetic constitution of an embryo and the environment in which it develops. Abnormalities in chromosomal division or gene mutations and other factors (e.g., mechanical, chemical, physical, and infectious agents) can interfere with embryo growth and differentiation and produce diverse effects depending on the teratogen involved and the developmental stage of the embryo at the time of exposure [1,2].

The neuroanatomic findings and distribution of lesions in this calf may be attributable to an inherited disorder, manifesting in malformation of the face and the CNS. Although an infectious agent or an environmental factor cannot be definitively ruled out in the present case, the features are not consistent with those correlated with these agents and previously described in calves [1,2,3]. Most of the environmental agents that are reported to be teratogenic for cattle, such as mechanical (e.g., intrauterine pressure) or chemical (e.g., poisonous plants, therapeutic drugs) determine mostly skeletal malformations but not neural defects [1,2,3]. Hydranencephaly, porencephaly, hydrocephalus, and cerebellar hypoplasia are the main malformations of the central nervous system due to bovine viral diarrhea virus (BVDV), Schmallenberg virus (SBV), blue tongue virus (BTV), Akabane virus (AKAV), or Aino virus (AV) [14]. Intrauterine infection of the fetus during the susceptible periods of development may cause these brain disorders, which in the case of SBV, AKAV and AV infections may be associated with deformity of the axial and appendicular skeleton (e.g., *arthrogryposis multiplex congenita*) [13].

Intrauterine Zika virus (ZIKV) infection in pregnant women is a cause of microcephaly and other serious brain anomalies that can be accompanied by findings consistent with craniofacial disproportion [25]. However, no natural transmission of ZIKV in non-human vertebrate populations has been demonstrated to date [26]. To our best knowledge, a similar association between such severe congenital abnormalities of the CNS and the craniofacial structures has never been reported in calves before and warrants further investigation.

## 4. Conclusions

In conclusion, this case report describes a particular complex of congenital abnormalities affecting both the central nervous system and the craniofacial structures in a calf, not easily to classify and trace to a single specific condition. The craniofacial abnormalities identified suggested a median cleft face syndrome, while the central nervous system changes had features consistent with both alobar holoprosencephaly and hydranencephaly. To the authors knowledge, a similar association between such congenital abnormalities has never been reported in calves. 

## Figures and Tables

**Table 1:** Neuropathological features of hydranencephaly, holoprosencephaly, and the brain of the calf described here.

Title	Hydranencephaly	Holoprosencephaly (Alobar Type)	Calf
Longitudinal cerebral fissure	Normal	Absent	Ventrally developed
Prosencephalon	Abnormal ^a^	Abnormal ^b^	Abnormal ^b^
Diencephalon	Normal	Abnormal ^c^	Abnormal
Mesencephalon, pons and cerebellum	Normal	Normal	Normal
Spinal cord	Normal/Abnormal	Normal	Abnormal
Third ventricle	Normal	Abnormal ^d^	Abnormal ^e^
Olfactory lobes and tracts	Present	Absent	Absent
Midline facial abnormalities	Absent	Absent/Present	Present
Ocular distance	Normal	Normal/Hypotelorism	Hypertelorism
Cranial cavity	Normal	Normal/Dome-shaped	Dome-shaped
Meningo-encephalocele	No	Possible	Yes (Meningocele)

^a^ Destroyed cerebral cortex, especially in its dorsal, medial, and caudal aspects, with enlarged lateral ventricles and with or without the absence of corpus callosum, septum pellucidum, rostral commissure, fornix, and hippocampus [2,4,5]. ^b^ Single telencephalic vesicle with the absence of corpus callosum, septum pellucidum, rostral commissure, fornix, and hippocampus and fused lateral ventricles [2,4,5]. ^c^ Incomplete hypothalamic and thalamic cleavage and partially or completely fused basal ganglia [2,4,5]. ^d^ The third ventricle forms a single ventricular cavity with the two lateral ventricles [2,4,5]. ^e^ Presence of multiple small ventricles within the diencephalon.
